# Altered pattern analysis and identification of subjective cognitive decline based on morphological brain network

**DOI:** 10.3389/fnagi.2022.965923

**Published:** 2022-08-11

**Authors:** Xiaowen Xu, Peiying Chen, Yongsheng Xiang, Zhongfeng Xie, Qiang Yu, Xiang Zhou, Peijun Wang

**Affiliations:** Department of Medical Imaging, Tongji Hospital, Tongji University School of Medicine, Tongji University, Shanghai, China

**Keywords:** subjective cognitive decline, structural magnetic resonance imaging, morphological brain network, graph theory, multiple kernel learning

## Abstract

Subjective cognitive decline (SCD) is considered the first stage of Alzheimer’s disease (AD). Accurate diagnosis and the exploration of the pathological mechanism of SCD are extremely valuable for targeted AD prevention. However, there is little knowledge of the specific altered morphological network patterns in SCD individuals. In this present study, 36 SCD cases and 34 paired-matched normal controls (NCs) were recruited. The Jensen-Shannon distance-based similarity (JSS) method was implemented to construct and derive the attributes of multiple brain connectomes (i.e., morphological brain connections and global and nodal graph metrics) of individual morphological brain networks. A t-test was used to discriminate between the selected nodal graph metrics, while the leave-one-out cross-validation (LOOCV) was used to obtain consensus connections. Comparisons were performed to explore the altered patterns of connectome features. Further, the multiple kernel support vector machine (MK-SVM) was used for combining brain connectomes and differentiating SCD from NCs. We showed that the consensus connections and nodal graph metrics with the most discriminative ability were mostly found in the frontal, limbic, and parietal lobes, corresponding to the default mode network (DMN) and frontoparietal task control (FTC) network. Altered pattern analysis demonstrated that SCD cases had a tendency for modularity and local efficiency enhancement. Additionally, using the MK-SVM to combine the features of multiple brain connectomes was associated with optimal classification performance [area under the curve (AUC): 0.9510, sensitivity: 97.22%, specificity: 85.29%, and accuracy: 91.43%]. Therefore, our study highlighted the combination of multiple connectome attributes based on morphological brain networks and offered a valuable method for distinguishing SCD individuals from NCs. Moreover, the altered patterns of multidimensional connectome attributes provided a promising insight into the neuroimaging mechanism and early intervention in SCD subjects.

## Introduction

Alzheimer’s disease (AD) is a neurodegenerative disease accompanied by cognitive decline, changes in personality, and impaired ability to perform daily activities. A total of 131.5 million people are estimated to have dementia by 2050 worldwide (Quinn et al., 2018). Early AD prevention and treatment are critical. Subjective cognitive decline (SCD), a self-experienced and reported worsening of confusion and memory loss, is one of the initial manifestations of preclinical AD (Jessen et al., 2014). Therefore, finding objective evidence to diagnose SCD early is extremely valuable for targeted AD prevention.

Researchers have used noninvasive magnetic resonance imaging (MRI) to assess alterations in brain structure and function in the initial asymptomatic stages of AD (Xu et al., 2021). Several studies have found that SCD patients had lower hippocampal volume and thinner cortical thickness in their temporoparietal lobe, which was linked to faster subsequent memory loss and higher risk of disease aggravation (Verfaillie et al., 2016, Verfaillie et al., 2018a; Yue et al., 2018). In addition, various MRI techniques, such as resting-state functional MRI (rs-fMRI), diffusion tensor imaging (DTI), and three-dimensional (3D) T1-weighted images (WI) structural MRI (3D-T1WI sMRI), have been used to assess changes in the morphology, structure, and function of brain network and provided new insights into the topological organization of graph theory attributes in SCD individuals. Furthermore, rs-fMRI has found that SCD cases had increased functional connectivity in their retrosplenial cortex and precuneus but decreased functional connectivity in their frontoparietal cortex and posterior memory system (Dillen et al., 2016; Dong et al., 2018; Viviano et al., 2019). In a study by Shu et al. (2018), the investigators used DTI to investigate the brain structural connectome in SCD patients and found significant disruptions in the topological efficiency in structural connectomes associated with memory impairment. Recently, the individual morphological brain network construction methods based on 3D-T1 sMRI have been used to explore the characteristics of brain networks (Kong et al., 2014). Some researchers (Tijms et al., 2018; Verfaillie et al., 2018b) have used the individual morphological brain network approach in SCD patients and found that they exhibited abnormal topological attributes, such as lower path length values in the precuneus, frontal, occipital, and temporal lobes, which were associated with disease progression and obvious deterioration in clinical cognitive performance. Nevertheless, the specific alterations in various topological properties of SCD patients and their value for early identification of SCD remain to be further investigated.

For the diverse connectome indicators derived from morphological brain networks, such as morphological brain connections and global and nodal graph metrics, a combination of multidimensional data was conducted to distinguishing the SCD individuals from normal controls (NCs). In our previous study, this proposed method, multiple kernel support vector machine (MK-SVM), was used to fuse the functional brain connectome information and has demonstrated a good classification performance in differentiating between patients with mild cognitive impairment (MCI) and NCs (Xu et al., 2020a). However, the combination of topological features of structural brain networks to accurately identify SCD patients remains to be further validated.

By combining graph-theoretic analysis and MK-SVM based on individual-level morphological brain network, this study primarily aimed to: (i) identify the discriminative topology properties and specific brain areas of SCD subjects; (ii) determine the distinctive alteration patterns in connectome features that are significantly different between SCD and NC groups; and (iii) explore an accurate classifier for distinguishing SCD patients from NCs.

## Materials and Methods

### Participants

For this study, 36 SCD patients and 34 NCs were selected. Each participant underwent neuropsychological and neuroimaging tests. The neuropsychological scales used in this study were the Verbal Fluency Test (VFT), Activity of Daily Living Scale (ADL), Auditory Verbal Learning Test (AVLT; Vakil and Blachstein, 1993), Geriatric Depression Scale (GDS; Sawada et al., 2019), and Montreal Cognitive Assessment (MoCA; Nasreddine et al., 2005). SCD cases were selected based on the following criteria (Jessen et al., 2014): (a) the age of onset of >60 years; (b) gradual decrease in self-perceived memory during the past 5 years (compared to initial non-disease state) or that could be validated by a close caregiver; (c) normal general cognitive function, as confirmed by the objective scale. NCs comprised participants with no cognitive impairment and normal neuropsychological scale scores.

The study protocol was approved by the Ethics Committee of Tongji Hospital of Tongji University (Shanghai, China). Before sample enrollment, each participant or their legal representative(s) provided signed consent for participation.

### Data acquisition

The 3.0T MagnetomVerio MRI scanner (Siemens, Munich, Germany), equipped with 32-channel head coils, was used to perform T1WI-MRI on each participant. During the MRI, each participant was advised and guided to: (1) close their eyes (not sleeping); (2) keep calm and avoid any thoughts as much as possible; and (3) avoid any movements. High-resolution T1WI 3D scans were obtained by using the 3D magnetization-prepared rapid gradient echo (MP-RAGE) at the following parameters: slice number = 192; flip angle = 7°; matrix size = 256 × 256; echo time (TE) = 2.98 ms; inversion time (TI) = 1,100 ms; repetition time (TR) = 2,530 ms; slice thickness = 1.0 mm; field of view (FOV) = 256 × 256 mm^2^, voxel size = 1.0 × 1.0 × 1.0 mm^3^. The scan was performed in 6.03 min.

### Preprocessing of MRI

Statistical Parametric Mapping (SPM12; Pataky, 2010) was used to preprocess the scan images. Voxel-based morphometric (VBM) was used to segment individual structural MRI images into the cerebrospinal fluid (CSF), gray matter (GM), and white matter (Ashburner and Friston, 2000). DARTEL was used to normalize the GM images according to the Montreal Neurologic Institute (MNI) criteria (Ashburner, 2007). Jacobian determinants were used to compensate and modify the effects of spatial normalization. Lastly, smoothing of all normalized T1WI structural images was performed using a 6.0-mm full width at half-maximum Gaussian kernel to enhance the signal-to-noise ratio and allow even data distribution (Shen and Sterr, 2013).

### Construction of individual morphological brain network

The brain was divided into 90 regions of interest (ROIs) based on the Automatic Anatomical Labeling (AAL) atlas, which were defined as nodes of the morphological brain network (Tzourio-Mazoyer et al., 2002). Jensen-Shannon distance-based similarity (JSS) was used to evaluate the morphological brain network connections between two brain ROIs (Endres and Schindelin, 2003; Peng et al., 2022). First, the GM volume values were extracted from all voxels of each brain region. Second, the kernel density estimation (KDE) was used for calculating the GM volume values probability density function. Third, the probability distribution function (PDF) was calculated for the derived GM volume value probability density function. Lastly, based on the probability distribution function, we calculated the JSS value between any pair of ROIs. The value range of JSS was (0, 1), where 1 represented the same distribution. Therefore, a closer GM density distribution between two ROIs was represented by a higher JSS value. The set of sparsity thresholds (range, 0.1–0.4; steps, 0.01) was also used for constructing an undirected binary network.

### Statistical analyses

The Statistical Package for Social Science (SPSS v26; IBM, Armonk, NY, USA) was used to compare baseline data between SCD patients and NCs with a two-sample t-test, rank-sum test, or chi-squared (χ^2^) test. A P value of <0.01 was used as the threshold for statistical difference. Comparisons between discriminative brain network connectome features were performed with the two-sample t-test (*P* < 0.01). Their false discovery rate for multiple comparisons was also determined for corrections as needed.

### Feature selection and classification

The t-test was applied to identify discriminative brain regions and nodal graph metrics of the brain network. For the high-dimensional connectome information, MK-SVM was used to combine different types of connectome features. The MATLAB LIBSVM toolbox3 was used to perform the MK-SVM classification (Xu et al., 2020a). The specific calculation process was listed as follows.


$$\underset{w}{\min}\frac{1}{2}\sum_{m=1}^{3}\beta_{m}∥w^{m}{∥}^{2}+C\sum_{i=1}^{n}\xi_{i}$$

$$s.t.y_{i}\left(\sum_{m=1}^{3}\beta_{m}{\left(w^{m}\right)}^{T}\phi^{m}\left(x_{i}^{m}\right)+b\right)\geq 1-\xi_{i}$$

$$\xi_{i}\geq 0,i=1,2,...,n$$

Here, *n* indicates the size of the sample, *x^1^_i_, x^2^_i_, and x^3^_i_* represent the value of brain connection, global metrics, and nodal graph metrics, respectively, of the *i*th sample where $k^{m}\left(x_{i}^{m},y_{i}^{m}\right)=\phi^{m}{\left(x_{i}^{m}\right)}^{T}\phi^{m}\left(x_{j}^{m}\right)$ is the kernel matrix for *m*th modality. The following equation can be used to represent the dual form of MK-SVM.


$$\underset{\alpha }{\max}\sum_{i=1}^{n}\alpha_{i}-\frac{1}{2}\sum_{i,j}\alpha_{i}\alpha_{j}y_{i}y_{j}\sum_{m=1}^{3}\beta_{m}k^{m}\left(x_{i}^{m},y_{i}^{m}\right)$$

$$s.t.\sum_{i=1}^{n}\alpha_{i}y_{i}=0$$

$$0\leq \alpha_{i}\leq C,i=1,2,...,n$$

Here, $k^{m}\left(x_{i}^{m},y_{i}^{m}\right)=\phi^{m}{\left(x_{i}^{m}\right)}^{T}\phi^{m}\left(x_{j}^{m}\right)$ represent the kernel matrix on the *m*th modality. $k^{m}\left(x_{i}^{m},x^{m}\right)=\phi^{m}{\left(x_{i}^{m}\right)}^{T}\phi^{m}\left(x^{m}\right)$ was used to define the kernel matrix between a new test sample and the *i*th training sample for the *m*th modality. MK-SVM was used to assess the classification performance using the following equation.


$$f\left(x_{1},x_{2},...,x_{m}\right)=sign\left(\sum_{i=1}^{n}y_{i}\alpha_{i}\sum_{m=1}^{M}\beta_{m}k^{m}\left(x_{i}^{m},x^{m}\right)+b\right)$$

*β*_m_ was used on the grid-searching space based on a cross-validation scheme using the constraint $\sum_{m}\beta_{m}=1.C$ ranged between 2^−5^ and 2^5^.

Considering the small sample size used in the present study, we utilized the Leave-one-out cross-validation (LOOCV) strategy to optimize the parameters and discern classification performance. The classification performance was compared for a single feature and the combination of different connectome features, including morphological brain connection (C), global graph metrics (G), nodal graph metrics (N), C+G+N, C+N, G+N, and C+G, respectively.

### Consensus connections

**Figure 1:**
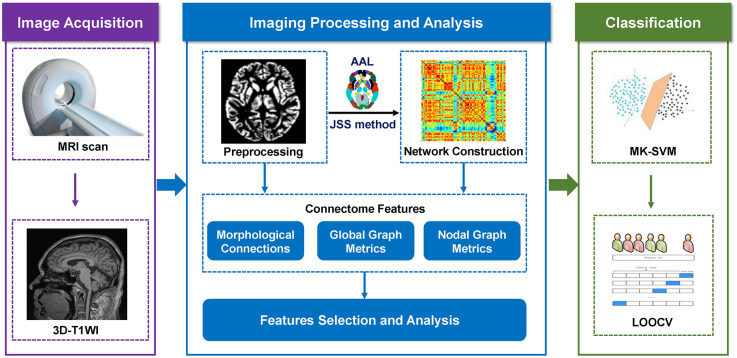
The procedure of data processing and classification.

When using the nested cross-validation strategy to assess the classification performance based on the proposed MK-SVM method, all selected connection features during the training process were recorded. In the present study, as the selected features by t-tests in each validation might be different, we recorded all the selected features during the training process. The consensus connections refer to the features that are consistently selected in all validations (Dosenbach et al., 2010; Zeng et al., 2012). Therefore, in our study, the consensus connections of brain networks were considered the most discriminative features to explore the pathological mechanisms and potential biomarkers associated with SCD. The data processing and classification methodologies are illustrated in Figure 1.

## Results

### Demographic and neurocognitive characteristics

Table 1 shows the characteristics of the study participants. Our findings showed that the SCD group had significantly higher VFT-vegetable scores than the NC group (*p* < 0.01). The other variables between the two groups were not significantly different.

**Table 1:** Demographic and neurocognitive characteristics of the NC and SCD groups.

**Variables**	**SCD (*n* = 36)**	**NC (*n* = 34)**	**T/X^2^/Z**	***P***
Age (years)	67.89 ± 6.395	69.24 ± 6.228	−1.065^c^	0.287
Education	11.19 ± 2.806	10.29 ± 2.970	1.321^c^	0.186
Gender (F/M)	27/9	20/14	2.074^b^	0.150
MoCA	24.25 ± 2.862	22.50 ± 3.612	−2.253^a^	0.027
Type of AVLT recall				
Immediate	17.83 ± 4.379	16.15 ± 4.083	1.686^c^	0.092
Short-delayed	6.06 ± 1.999	5.35 ± 2.581	−1.278^a^	0.206
Long-delayed	5.61 ± 2.533	4.09 ± 2.906	2.069^c^	0.039
VFT-vegetable^*^	16.50 ± 3.946	14.00 ± 3.104	−2.955^a^	0.004
VFT-fruit	11.47 ± 3.229	11.38 ± 2.871	−0.123^a^	0.903
VFT-idiom	4.92 ± 3.865	3.53 ± 3.277	1.434^c^	0.152
GDS	4.03 ± 4.766	4.85 ± 6.629	0.595^c^	0.552
ADL	14.08 ± 0.280	15.00 ± 2.934	−1.651^c^	0.099

^*^P < 0.01, significant differences between the two groups. ^a^T, derived from the two-sample t-test. ^b^X^2^, derived from the chi-square test. ^c^Z, derived from the rank-sum test. The data represent the mean ± standard deviation (SD). SCD, subjective cognitive decline; NC, normal control; MoCA, Montreal Cognitive Assessment; AVLT, Auditory Verbal Learning Test; VFT, Verbal Fluency Test; GDS, Geriatric Depression Scale; ADL, Activity of Daily Living Scale.

### Graph metrics of the morphological brain connectome

**Figure 2:**
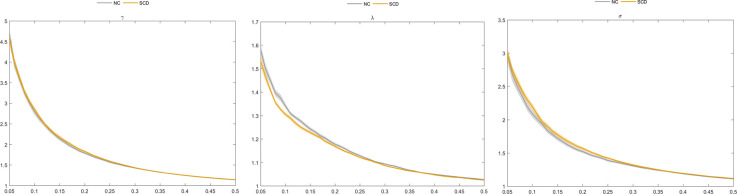
Comparison of normalized clustering coefficient (γ), normalized characteristic path length (λ), and “small world” (σ) between the subjective cognitive decline (SCD) and normal control (NC) groups.

Our results showed that an increase in the connection density was associated with an increase in the value of C_p_, E_global_, and E_local_ and a decrease in L_p_, λ, γ, σ, and Q between the two groups (Table 2; Figure 2). Moreover, SCD patients and NCs both fitted γ = C_p_^real^/C_p_^rand^>1, and λ = L_p_^real^/L_p_^rand^≈1, indicating that the morphological networks of the brain were associated with greater real C_p_ values and similar real L_p_ values compared with the matched random networks. Thus, both groups met the “small-world” topological attributes. Further, we observed that SCD patients had lower λ values than NCs for almost the entire range of connection density (*P* < 0.01).

**Table 2:** Statistical result of graph metrics between the two groups.

**Global graph metrics**	**SCD**	**NC**
*C*_p_	0.2946 ± 0.01	0.2949 ± 0.01
*L*_p_	0.9445 ± 0.02	0.9557 ± 0.03
γ	0.8123 ± 0.06	0.8044 ± 0.07
λ^*^	0.5152 ± 0.01	0.5203 ± 0.01
σ	0.6899 ± 0.05	0.6762 ± 0.06
*E*_global_	0.2375 ± 0.00	0.2358 ± 0.01
*E*_local_	0.3502 ± 0.01	0.3497 ± 0.01
*Q*	13.7664 ± 1.05	13.5253 ± 1.12

C_p_, clustering coefficient; *E_global_*, global efficiency; *E_local_*, local efficiency; *L_p_*, characteristic path length; NC, normal control; SCD, subjective cognitive decline; Q, modularity score; γ, normalized clustering coefficient; λ, normalized characteristic path length; σ, small world. ^*^Significant with FDR (0.05).

We analyzed the most discriminative nodal graph topological features. Table 3 shows that the betweenness centrality, degree centrality, and nodal efficiency were the most discriminative abilities between the two groups and were mostly found in the limbic and frontal lobes, corresponding to DMN, frontoparietal task control (FTC), and sensory/somatomotor hand (SH) brain networks. Further comparisons of nodal graph metrics revealed that SCD patients had significantly higher values of betweenness centrality, nodal clustering coefficient, degree centrality, nodal efficiency, and local nodal efficiency in the frontal lobe (e.g., the bilateral inferior frontal and right superior frontal gyri), limbic lobe (e.g., left parahippocampal gyri), and central region (e.g., left postcentral gyri). In contrast, the nodal shortest path length and degree centrality values were significantly lower in the left supplementary motor area, left superior frontal gyri, and medial orbital gyri (*P* < 0.01 for all).

**Table 3:** Top 15 most discriminative nodal graph metrics.

**Nodal graph metrics**	**Mean value**		**AAL brain regions**	**Sub-network**
	**SCD**	**NC**		
Nodal efficiency	0.290	0.273	PHG.L	DMN
Degree centrality	16.711	14.391	PHG.L	DMN
Betweenness centrality	29.788	20.937	ORBinf.R	DMN
Nodal efficiency	0.260	0.244	IFGoperc.R	FTC
Nodal clustering coefficient	0.318	0.287	PoCG.L	SH
Betweenness centrality	41.295	31.871	PHG.L	DMN
Degree centrality	12.434	10.691	IFGoperc.R	FTC
Nodal efficiency	0.296	0.280	IFGtriang.L	FTC
Nodal local efficiency	0.376	0.344	PoCG.L	SH
Nodal efficiency	0.278	0.260	SFGdor.R	DMN
Nodal shortest path	0.811	1.381	SMA.L	CTC
Degree centrality	17.332	15.560	IFGtriang.L	FTC
Degree centrality	14.810	12.909	SFGdor.R	DMN
Degree centrality	9.247	11.050	ORBsupmed.L	DMN
Nodal efficiency	0.253	0.231	ORBsup.R	FTC

AAL, automated anatomical labeling atlas; DMN, default mode network; FTC, frontoparietal task control; SH, sensory/somatomotor hand; CTC, cingulo-opercular task control.

### Consensus connections of morphological brain connectome

**Figure 3:**
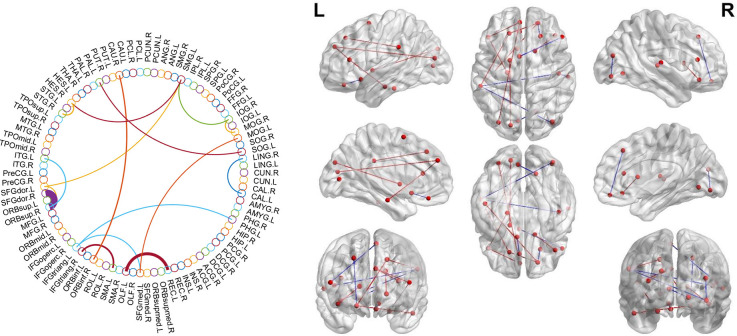
Theconsensus connections of the morphological brain network. Left: theconsensus connections of the morphological brain network selected byleave-one-out cross-validation (LOOCV) in the subjective cognitivedecline (SCD) and normal control (NC) groups based on AAL90. Thethickness of an arc in the circle indicates the discriminative powerof an edge, which is inversely proportional to the estimatedP-values. The colors were randomly generated to differentiate regionsof interest (ROIs). Right: the consensus connections selected by LOOCV. The connections were mapped on the ICBM 152 template with the BrainNetViewer package (http://nitrc.org/projects/bnv/). Blue and red represent the decrease and increase of morphological connection weight of SCD groups, respectively.

We investigated the significantly different consensus connections between the two groups. As shown in Table 4 and Figure 3; most of the consensus connections were found in the frontal, temporal, parietal, and occipital lobes. Furthermore, corresponding subnetworks to these brain regions were the DMN, visual network, and auditory network. Additionally, our results suggested that the mean values of consensus connections distributed in the occipital and temporal lobes were lower in the SCD group than in the NC group. However, the mean values of consensus connections distributed in the frontal lobe or between the frontal lobe and other brain regions (e.g., the temporal and parietal lobes) of SCD patients were higher than those in NCs.

**Table 4:** Consensus connections in the NC and SCD groups.

**ROI**	**ROI**	**Mean value**		**P**
		**SCD**	**NC**	
REC.L	OLF.L	0.392	0.642	9.340 × 10^−4^
CAU.R	ORBinf.R	0.704	0.463	3.655 × 10^−3^
HES.R	STG.R	0.173	0.289	3.779 × 10^−3^
ITG.L	ORBsup.R	0.770	0.526	4.928 × 10^−3^
MOG.R	IOG.R	0.461	1.133	5.570 × 10^−3^
PHG.L	IFGtriang.L	1.429	0.925	6.198 × 10^−3^
SFGmed.L	IFGtriang.L	0.937	0.877	6.545 × 10^−3^
SMG.L	IOG.R	0.594	0.998	7.378 × 10^−3^
LING.R	CAL.L	0.856	1.052	7.451 × 10^−3^
SMG.L	SFGdor.L	1.523	0.672	7.968 × 10^−3^
SFGmed.L	MOG.L	1.105	0.834	8.223 × 10^−3^

ROI, region of interest.

### Classification

**Figure 4:**
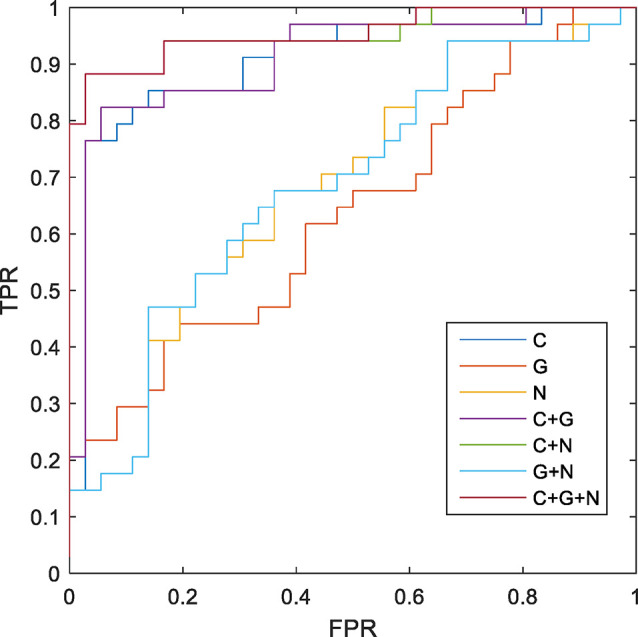
Receiver operating characteristic (ROC) of classification based on different morphological connectome features. C, connection; G, global metrics; N, nodal metrics; FPR, false-positive rate; TPR, true-positive rate.

The MK-SVM method was used to differentiate SCD patients from NCs based on brain connectome information (Table 5; Figure 4). Our results showed that the classification accuracy of the brain networks C, G, and N was 82.86%, 55.71%, and 61.43%, respectively. Furthermore, we performed combinations of the brain network graph metrics, and our results showed that the classification accuracy for C+G, C+N, and G+N was 84.29%, 90.00%, and 62.85%, respectively. The optimal classification performance was with the combination of C, N, and G, which demonstrated an accuracy, sensitivity, specificity, and area under the curve (AUC) of 91.43%, 97.22%, 85.29%, and 0.9510, respectively, indicating that combining the multimodal features could effectively boost the performance of the classification.

**Table 5:** Classification performance of different structural graph metrics.

**Method**	**Accuracy (%)**	**Sensitivity (%)**	**Specificity (%)**	**AUC**
C	82.86	88.89	76.47	0.9027
G	55.71	61.11	50.00	0.6266
N	61.43	69.44	52.94	0.6756
MK_CG	84.29	88.89	79.41	0.9061
MK_CN	90.00	97.22	82.35	0.9509
MK_GN	62.85	72.22	52.94	0.6781
MK_CGN	91.43	97.22	85.29	0.9510

Structural connectivity (C), Global metric (G), Nodal metric (N); MK-SVM, multiple kernel support vector machine.

## Discussion

Compared with previous morphological brain network construction methods, the JSS method provides a more accurate evaluation of the similarity between brain regions (Endres and Schindelin, 2003; Li et al., 2021). The quantitative and symmetric JSS divergence evaluation method enables a more objective and accurate description of connections between brain regions in morphological brain networks. Based on the individual morphological brain network, we could identify the most significantly affected brain regions and specific graph metrics that could differentiate between SCD patients and NCs. The altered patterns of topological properties of the morphological brain connectome indicated the enhancement of local brain network function associated with SCD. We trained a classifier for differentiating SCD patients from NCs and acquired a surprising result based on the MK-SVM method. Finally, we applied the MK-SVM method for the combination of multidimensional brain network connectome features and distinguishing SCD patients from NCs.

### Most discriminative brain network features and the altered patterns

For the discriminative brain network features identified in this study, our results indicated that the nodal graph metrics and consensus connections with the most discriminative abilities were primarily located in the frontal, limbic, and parietal lobes. Furthermore, an analysis showed that when these brain regions were projected to subnetworks, most of them were located in the DMN and FTC networks, among which DMN demonstrated the greatest ability to distinguish SCD patients from NCs. Previous literature has reported an association between DMN and episodic memory loss, which was then referred to as the most significant cognitive domain impairment in early-stage AD (Wang et al., 2013; Joshi et al., 2019). Previous studies comparing the functional brain networks between SCD and MCI patients have also confirmed these early alterations and the important role of DMN-related brain regions (Xu et al., 2020a, b). Nevertheless, our study validated the significant changes in DMN in SCD patients from the individual morphological brain network perspective. Thus, our results not only validated the discriminative ability of the DMN for discriminating NCs from SCD patients but also showed the repeatability and verifiability of the proposed methods.

For the altered pattern analysis of brain network connectome features, our results showed that both SCD patients and NCs met the “small-world” topological attributes, which was consistent with our previous findings on functional brain networks. This suggests the high efficiency of brain networking in integrating information rapidly in real time across brain regions to actively optimize information processing between brain regions at the lowest cost possible (Watts and Strogatz, 1998; Liao et al., 2017). In addition, some previous studies based on the white matter structural network or functional network have found that functional integration among brain regions was decreased in SCD (Xu et al., 2020b; Tao et al., 2021). In our study, we found that a decreased value of λ was associated with an increase in the function of brain network integration in SCD, suggesting that SCD patients had an enhanced ability to communicate and transmit global information compared to NCs, which might be related to compensatory alterations in the morphological brain networks during SCD progression.

At the level of local brain regions, we observed that the discriminative ability for nodal efficiency, degree centrality, and betweenness centrality was the most significant among the selected nodal graph measurements. A previous study has found that SCD patients had less global efficiency and local efficiency mainly distributed in the bilateral prefrontal regions and left thalamus and demonstrated the disruption of structural network topology in SCD (Shu et al., 2018). In our study, an increase in nodal clustering coefficient and local efficiency and reduced values in nodal shortest path length in the frontal and limbic lobes were associated with increased modularity and local efficiency in the morphological brain network in SCD patients. The result reflected the enhanced function of brain network segregation in SCD, which were also found in functional brain network (Xu et al., 2020b). Similarly, the increased mean value of consensus connections distributed between the frontal lobe or frontal lobes and other brain regions (e.g., the temporal and parietal lobes) in the SCD group indicated the compensatory changes in the morphological brain network with enhanced connectivity of some brain regions. As mentioned in a previous study, these compensatory changes may be attributed to the indistinctive decline in cognitive function during SCD (Chen et al., 2020).

### Classification performance of different connectome features

This study used MK-SVM to combine brain connectomes and differentiate SCD patients from NCs. MK-SVM is a sparse machine-learning method that can solve imbalanced dimension issues to achieve the best classification performance. As shown in Table 5, brain connections exhibited the most excellent performance for the single modality of morphological brain network connectome, with an AUC of 0.9027. We found that regardless of global or nodal graph metrics, combining with brain connections could effectively improve their classification performance, the reason for which may be that brain connections carry abundant information. Notably, despite the worst classification performance of the global graph metrics among these features, it does not mean that it is insignificant, and the result may be related to the low dimension of the data it contains. The global graph metrics were very meaningful for exploring the global properties of brain network connectomes and disease mechanisms. Although the AUC of global and nodal graph metrics were lower than brain connections, the combination of C+G, C+N, and G+N significantly improved classification performance. Particularly, combining three brain connectome features (C+G+N) with MK-SVM demonstrated optimal classification performance, with an AUC of 0.9510, which was superior to the SCD classification based on the functional brain network in our previous study (Xu et al., 2020b). Recent studies, such as that by Huang et al. (2021) have employed MK-SVM to integrate information from three types of white matter networks and obtained an accuracy of 83.3% for distinguishing MCI subjects from NCs. Previous researchers, using the linear kernel SVM, achieved the accuracy of 79.49% and 83.13% in two different cohorts for the diagnosis of SCD individuals (Lin et al., 2022). For our results, these results can only be used for reference and compared to some extent. Due to the differences in data sets and model parameters, further verification is needed to understand whether different classification models can be compared or not. In sum, our results demonstrated that a combination of brain connectome features provided complementary information to each other and further enhanced SCD classification performance.

### Limitations and perspectives

There were some limitations to the present study that need to be addressed in the future. First, it was a single-center study with a relatively small number of participants. Hence, the robustness and generalizability of the proposed model still require further validation and improvement in multicenter and larger cohort studies. Second, only neuroimaging information was used in this study, but we intend to combine SCD with additional diagnostic tests (i.e., PET, electroencephalography, biomarkers, and clinical cognitive function examinations) to deepen our understanding of SCD pathogenesis. Third, a stringent and longer follow-up of different AD stages *via* imaging techniques would be useful to identify early and specific markers that could improve the diagnosis of AD and predict its progression.

## Conclusions

This study showed that compared with NC, the most discriminative traits of SCD patients were located in the frontal, limbic, and parietal lobes, corresponding to DMN and FTC networks. The altered pattern analysis demonstrated that SCD was more inclined to modularity along with local efficiency enhancement. Furthermore, MK-SVM combined with multiple brain connectome features to overcome the problems of the high-dimensional curves and small samples and effectively improved the classification performance for SCD diagnosis. Our research findings provided insights for improving the SCD diagnosis. Multidimensional connectome attributes analysis based on the morphological changes in brain networks provides a promising approach for insight into the neuroimaging mechanism and early intervention in SCD subjects.

## Data Availability Statement

The original contributions presented in the study are included in the article, further inquiries can be directed to the corresponding author.

## Ethics Statement

The studies involving human participants were reviewed and approved and the study protocol was approved by the Ethics Committee of Tongji Hospital of Tongji University (Shanghai, China). The patients/participants provided their written informed consent to participate in this study.

## Author Contributions

Study design and writing: XX and PC. MRI scanning: YX. Diagnosis and data collection: ZX, QY, and XZ. Data analysis: XX and PC. Critical revision: PW. All authors contributed to the article and approved the submitted version.

## Funding

This work was partially supported by the National Natural Science Foundation of China (Grant Nos. 82102023, 81830059); the Clinical Research Plan of SHDC (No. SHDC2020CR1038B); Science and Technology Commission of Shanghai Municipality (No. 19411951400).
